# Modulation of Macrophages TLR4-Mediated Transcriptional Response by *Lacticaseibacillus rhamnosus* CRL1505 and *Lactiplantibacillus plantarum* CRL1506

**DOI:** 10.3390/ijms26062688

**Published:** 2025-03-17

**Authors:** Masahiko Suzuki, Ayelen Baillo, Leonardo Albarracin, Mariano Elean, Rodrigo Serda, Yoshihito Suda, Fu Namai, Keita Nishiyama, Haruki Kitazawa, Julio Villena

**Affiliations:** 1 Food and Feed Immunology Group, Laboratory of Animal Food Function, Graduate School of Agricultural Science, Tohoku University, Sendai 980-8572, Japan; masa_joe147@yahoo.co.jp (M.S.); fu.namai.a3@tohoku.ac.jp (F.N.); keita.nishiyama.a6@tohoku.ac.jp (K.N.); 2 Laboratory of Technology, Reference Centre for Lactobacilli (CERELA-CONICET), San Miguel de Tucumán 4000, Argentina; antobaillo22@gmail.com; 3 Laboratory of Immunobiotechnology, Reference Centre for Lactobacilli (CERELA-CONICET), San Miguel de Tucumán 4000, Argentina; lalbarracin@herrera.unt.edu.ar (L.A.); melean@cerela.org.ar (M.E.); rodrigo.serda@gmail.com (R.S.); 4 Department of Food, Agriculture and Environment, Miyagi University, Sendai 980-8572, Japan; suda@myu.ac.jp; 5 Livestock Immunology Unit, International Education and Research Centre for Food and Agricultural Immunology (CFAI), Graduate School of Agricultural Science, Tohoku University, Sendai 980-8572, Japan

**Keywords:** probiotics, macrophages, TLR4, *L. rhamnosus* CRL1505, *L. plantarum* CRL1506, transcriptomics

## Abstract

*Lacticaseibacillus rhamnosus* CRL1505 and *Lactiplantibacillus plantarum* CRL1506 increase the resistance of mice to Gram-negative pathogens infections. In this work, we advanced the characterization of the CRL1505 and CRL1506 immunomodulatory properties by evaluating their effect on the Toll-like receptor 4 (TLR4)-triggered immune response in macrophages. We performed experiments in murine RAW 264.7 macrophages stimulated with lipopolysaccharide (LPS) to evaluate the transcriptomic changes induced by lactobacilli. These in vitro experiments were complemented with in vivo studies in mice to determine the effect of CRL1505 and CRL1506 strains on Peyer’s patches and peritoneal macrophages. Microarray transcriptomic studies and qPCR confirmation showed that the CRL1505 and CRL1506 strains modulated the expression of inflammatory cytokines and chemokines as well as adhesion molecules in LPS-challenged RAW macrophages, making the effect of *L. rhamnosus* CRL1505 more remarkable. Lactobacilli also modulate regulatory factors in macrophages. *L. plantarum* CRL1506 increased *il10* and *socs2* while *L. rhamnosus* CRL1505 upregulated *il27*, *socs1*, and *socs3* in RAW cells, indicating a strain-specific effect. However, in vivo, both strains induced similar effects. Peyer’s patches and peritoneal macrophages from mice treated with lactobacilli produced higher levels of tumor necrosis factor (TNF)-α, interferon (IFN)-γ, interleukin (IL)-6, and colony stimulating factor (CSF)-3 after LPS stimulation. This effect would allow improved protection against pathogens. In addition, both lactobacilli equally modulated *socs1* and *socs2* expressions and IL-10 and IL-27 production in Peyer’s patches macrophages and *socs3* and IL-10 in peritoneal cells. Furthermore, lactobacilli reduced the production of IL-1β, IL-12, CSF2, C-C motif chemokine ligand (CCL)-2, and CCL8 in LPS-challenged macrophages. This differential modulation of regulatory and inflammatory factors would allow minimal inflammatory-mediated tissue damage during the generation of the innate immune response. This work provides evidence that *L. rhamnosus* CRL1505 and *L. plantarum* CRL1506 modulate macrophages’ TLR4-mediated immunotranscriptomic response, helping to improve protection against Gram-negative bacterial infections.

## 1. Introduction

Gram-negative bacteria, particularly those belonging to the *Enterobacteriacacae* family, including *Salmonella*, *Citrobacter*, *Shigella*, and pathogenic *Escherichia*, induce severe diarrhea and systemic disease through the damage of the intestinal mucosa [1]. This damage can be triggered by their virulence factors as well as by the induction of a host’s deregulated inflammatory response [1]. The intestinal innate immune system detects the presence of Gram-negative pathogens through the interactions of the microbial-associated molecular patterns (MAMPs) with pattern recognition receptors (PRRs) expressed in intestinal epithelial cells (IECs) and tissue-resident immune cells [2]. The MAMPs-PRRs interaction conducts the activation of nuclear factor κB (NF-κB), mitogen-activated protein kinase (MAPK), and interferon regulatory factor-3 (IRF3) signaling pathways, leading to the production of antimicrobial peptides, inflammatory cytokines and chemokines, and type I interferons (IFNs) that mediate the clearance of pathogens. The generation of inflammatory responses in the intestinal mucosa in front of the pathogen’s attacks is necessary to eliminate the invading microorganisms. However, if this innate line of defense is dysregulated or prolonged, it may lead to intestinal damage and dysfunction [3]. The infection of the intestinal epithelium by Gram-negative pathogens induces the production of potent inflammatory mediators like interleukin (IL)-1β and IL-18 that induce the recruitment and activation of neutrophils and monocytes that collaborate in the elimination of pathogens like *Shigella* [4] and *Salmonella* [5]. On the other hand, several studies have shown that the dysregulated activation of the Toll-like receptor 4 (TLR4) by the Gram-negative MAMP lipopolysaccharide (LPS) decreases the villus height and alters the expression of the genes associated with the intestinal barrier function through the induction of inflammatory factors including tumor necrosis factor-α (TNF-α), IL-1β, and IL-6 [6,7]. These studies show that the appropriate production of inflammatory cytokines and chemokines and their regulation by anti-inflammatory mediators like IL-10 and IL-27 is of crucial importance for eliminating pathogens with minimal damage to the host.

The intestine is a complex heterogeneous tissue with different populations of macrophages that occupy microanatomical niches and adapt their function according to the surrounding microenvironment [8]. Macrophages within the gut have a crucial function in the generation of effector responses that protect the intestinal tissue against pathogens with minimal inflammatory damage. TLR4 pathway activation in macrophages significantly contributes to the production of inflammatory mediators that coordinate the pathogen’s clearance. Macrophages also produce anti-inflammatory factors that help to control inflammation and participate in the healing of damaged tissue [2,8]. Then, the regulation of inflammatory and anti-inflammatory factors by activated macrophages during intestinal infections has been considered a therapeutic target for improving resistance to infections and protecting the host against inflammatory-related damage [9]. In this regard, beneficial microbes with immunomodulatory capacities were proposed as modulators for improving the resistance against Gram-negative pathogens through the regulation of TLR4-mediated innate immune response [10,11]. Studies with different immunomodulatory lactobacilli demonstrated that beneficial bacteria could modulate the cytokines produced by macrophages in a strain-dependent manner. Using the murine RAW 264.7 macrophage cell line, it was demonstrated consistently that probiotic lactobacilli can regulate the expression of inflammatory and regulatory cytokines in response to LPS stimulation. *Latilactobacillus sakei* CNS001WB and *Lactobacillus pentosus* WB693 downregulated the expressions of *tnfα*, *il6*, and *il1β* in RAW macrophages stimulated with LPS [12]. Similarly, *Lactiplantibacillus plantarum* LRCC5314 diminished the synthesis of IFN-γ, TNF-α, IL-6, and IL-1β [13]. It was also reported that *Lacticaseibacillus rhamnosus* 4B15 or *Lactobacillus gasseri* 4M13 significantly reduced the production of TNF-α, IL-6, and IL-1β [14]. *Lactobacillus helveticus* NS8 reduced *il12* [15], and *Lactiplantibacillus plantarum* LOC1 diminished the expression of the inflammatory factors *il1β*, *il12*, *csf2*, *cxcl3*, *cxcl1*, and *cx3cl1*, while augmenting *tnfα*, *il6*, *ifnγ*, *il15*, *cxcl9*, and *csf3* in RAW macrophages after the activation of TLR4 pathway [16]. Of note, *L. rhamnosus* 4B15 and *L. gasseri* 4M13 diminished the production of IL-10 [14], and *L. helveticus* NS8 augmented *il10* expression [15] while *L. plantarum* LOC1 induced no effect on *il10* but augmented *il27* expression [16]. Similar findings were described in experiments in which primary cultures of Peyer’s patches or peritoneal macrophages were used to evaluate the effect of lactobacilli on the TLR4 pathway [17,18]. These studies show the importance of macrophages in the beneficial modulation induced by lactobacilli on the immune responses against intestinal pathogens and highlight the strain-dependent effect. Thus, each immunomodulatory lactobacilli must be specifically evaluated in terms of its interaction with macrophages since extrapolations cannot be made with other strains, even from the same species.

*Lacticaseibacillus rhamnosus* CRL1505 and *Lactiplantibacillus plantarum* CRL1506 are two immunomodulatory strains with the ability to increase resistance against Gram-negative pathogens. We demonstrated previously that the oral administration of *L. rhamnosus* CRL1505 or *L. plantarum* CRL1506 improved the protection against *Salmonella enterica* subsp. *enterica* serovar Typhimurium infection in mice [19]. The CRL1505 and CRL1506 strains diminished the *Salmonella* counts in the liver and spleen and reduced the intestinal damage, effects that were associated with the induction of improved levels of TNF-α, IL-1β, IFN-γ, and IL-10 in the intestinal mucosa and serum. Similar results were obtained in a mice model of enterotoxigenic *E. coli* (ETEC) infection. Animals receiving the CRL1506 strain had significantly lower percentages of bodyweight loss, ETEC counts in jejunum and ileum, and intestinal tissue damage than control mice [20]. Furthermore, *L. plantarum* CRL1506 avoided the spread of ETEC to the liver, spleen, and blood. The protective effect conferred by the CRL1506 strain was associated with reduced levels of TNF-α, the functional IL-8 homolog KC and monocyte chemoattractant protein-1 (MCP-1) and increased production of IFN-γ and IL-10 in the intestinal mucosa. Studies performed in porcine intestinal epithelial cells (PIE cells) demonstrated that *L. plantarum* CRL1506 is able to differentially modulate the expression of inflammatory cytokines and chemokines after the activation of TLR4 [20]. PIE cells treated with the CRL1506 strain had significantly higher expression levels of *il6* and *ccl8* and lower *il8*, *ccl2*, *cxcl5*, *cxcl9*, and *cxcl11* than controls. This effect was related to the capacity of the CRL1506 strain to modulate the expression of the negative regulators of the TLR4 signaling as shown by the reductions in *tnfaip3* (A20) and *bcl3*, and the increase of *mkp1*. In addition, the ex vivo evaluation of phagocytic and bactericidal activities as well as cytokine production carried out in peritoneal macrophages isolated from mice treated with *L. plantarum* CRL1506 showed that this strain was capable of increasing macrophages’ phagocytic activity and their capacity to produce IFN-γ in response to LPS stimulation [20]. The modulation of these parameters in peritoneal macrophages was also observed after the oral treatment of mice with *L. rhamnosus* CRL1505 [21]. These previous results suggest that CRL1505 and CRL1506 strains could modulate the transcriptional response of intestinal and peritoneal macrophages in the context of TLR4 activation, improving the resistance to Gram-negative pathogens infections.

While previous studies suggest that CRL1505 and CRL1506 strains influence macrophage responses, the precise transcriptional mechanisms involved remain unclear. This study aims to bridge that knowledge gap by evaluating the effect of *L. rhamnosus* CRL1505 and *L. plantarum* CRL1506 on macrophages in the context of TLR4-mediated inflammation. We conducted in vitro studies in murine RAW macrophages to evaluate the ability of the lactobacilli strains to modulate the transcriptomic response after LPS stimulation. In addition, we performed experiments in mice to demonstrate in vivo the effect of CRL1505 and CRL1506 strains on Peyer’s patches and peritoneal macrophages’ ability to produce cytokines and chemokines in response to TLR4 activation.

## 2. Results

### 2.1. Effect of L. rhamnosus CRL1505 and L. plantarum CRL1506 on Macrophages TLR4-Mediated Transcriptomic Response

#### 2.1.1. The CRL1505 and CRL1506 Strains Modulate Immune Factors in LPS-Stimulated RAW Macrophages

**Figure 1:**
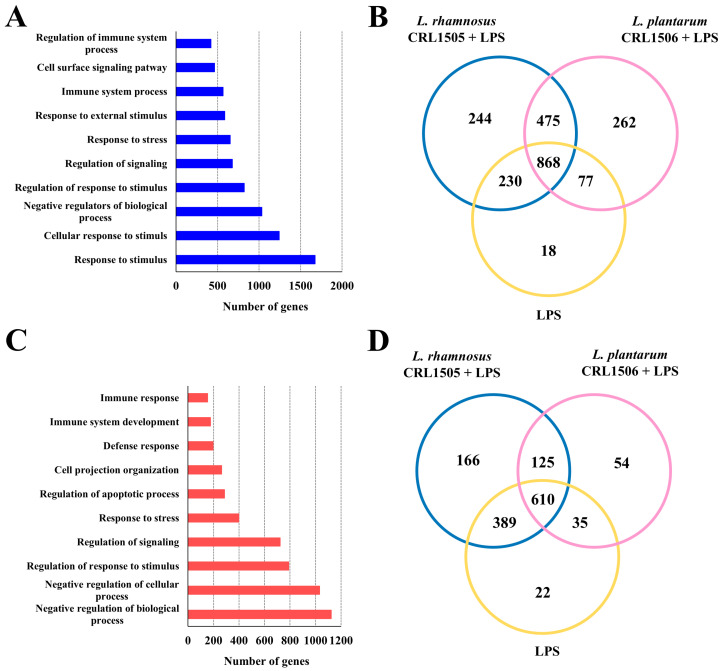
Effect of lactobacilli strains in murine macrophages (RAW cells) stimulated with the Toll-like receptor 4 (TLR4) agonist lipopolysaccharide (LPS). Macrophages were treated with *Lacticaseibacillus rhamnosus* CRL1505 or *Lactiplantibacillus plantarum* CRL1506 strains and then challenged with LPS. The expression of genes was determined by microarray analysis 12 h after LPS stimulation. Non-lactobacilli-treated macrophages stimulated with LPS were used as control. The numbers of up-regulated (**A**) and down-regulated (**C**) genes in the different categories of the Gene Ontology (GO) database. Venn diagrams show the number of differentially up-regulated (**B**) and down-regulated (**D**) genes for each experimental group.

The transcriptomic response of murine macrophages to the stimulation with the TLR4 agonist LPS as well as the impact of the immunomodulatory lactobacilli *L. rhamnosus* CRL1505 and *L. plantarum* CRL1506 on macrophage’s response was evaluated by microarray analysis. When the transcripts of macrophages stimulated with LPS were contrasted with the ones in non-stimulated cells it was observed that there were 2174 unique genes (Figure 1A,B) and 1401 unique genes (Figure 1C,D) up-regulated and down-regulated, respectively. Among the upregulated genes, most of them belonged to the GO Biological Process pathways “response to stimulus”, “cellular response to stimulus” and “negative regulators of biological processes” (Figure 1A) while most of the downregulated genes belonged to the categories of “negative regulators of biologicals processes”, “negative regulation of cellular biologicals processes” and “regulation of response to stimulus” (Figure 1C).

Of note, it was observed that CRL1505- and CRL1506-treated RAW macrophages had a differential expression in response to LPS stimulation compared to controls. Although the three groups had 868 upregulated genes, macrophages treated with the CRL1505 strain had 244 unique genes upregulated while those receiving the CRL1506 strain had 262 unique genes upregulated (Figure 1B). Furthermore, 18 upregulated genes were detected only in control LPS-challenged macrophages. Similarly, the three groups shared 610 downregulated genes, while macrophages treated with the CRL1505 or the CRL1506 strains had 166 and 54 unique genes downregulated, respectively (Figure 1D).

**Figure 2:**
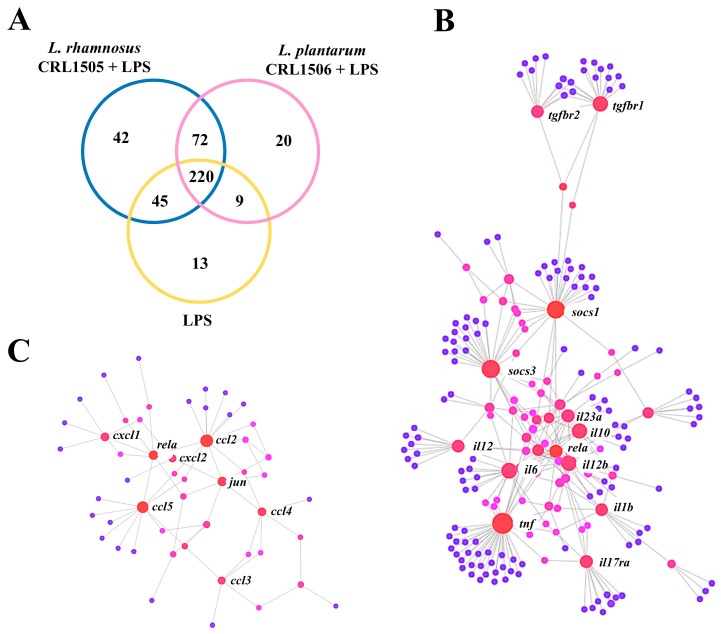
Effect of lactobacilli strains in murine macrophages (RAW cells) stimulated with the Toll-like receptor 4 (TLR4) agonist lipopolysaccharide (LPS). Macrophages were treated with *Lacticaseibacillus rhamnosus* CRL1505 or *Lactiplantibacillus plantarum* CRL1506 strains and then challenged with LPS. The expression of genes was determined by microarray analysis 12 h after LPS stimulation. Non-lactobacilli-treated macrophages stimulated with LPS were used as control. The Venn diagram shows the number of differentially regulated genes with immune-related functions for each experimental group (**A**). Protein–protein interaction network of the genes related to cytokines (**B**) and chemokines (**C**) pathways.

Out of the 3575 differentially regulated genes (Figure 1), 421 were assigned to immune-related functions according to the GO database (Figure 2A). It was also observed that CRL1505- and CRL1506-treated RAW macrophages had a differential expression of immune-related genes compared to controls. Although the three groups had 220 differentially regulated genes, macrophages treated with the CRL1505 and the CRL1506 strains had 42 and 20 unique genes (Figure 2A). The network analysis of differentially expressed genes revealed that the most remarkable changes in the transcriptomic response of macrophages after the LPS challenge were in the expression of cytokines (Figure 2B) and chemokines (Figure 2C) genes. Although both immunomodulatory lactobacilli are able to modify the transcriptomic response of RAW macrophages after LPS stimulation, the results showed that *L. rhamnosus* CRL1505 had a more remarkable effect than *L. plantarum* CRL1506.

Protein–protein interactions analysis among the differentially expressed immune genes detected central roles for *tnf*, *il1b*, *il6*, *il12*, *il10*, *il17ra*, *soc1*, *soc2*, and *tgfb* for cytokines (Figure 2B) and *rela*, *ccl2*, *ccl3*, *ccl4*, *ccl5*, *cxcl1*, *cxcl2*, and *jun* for chemokines (Figure 2C). Considering these results, we focused further analysis on the differential expression of cytokines and chemokines in LPS-challenged macrophages and the influence of immunomodulatory lactobacilli on those genes.

#### 2.1.2. The CRL1505 and CRL1506 Strains Modulate Inflammatory and Regulatory Cytokines in LPS-Stimulated RAW Macrophages

**Figure 3:**
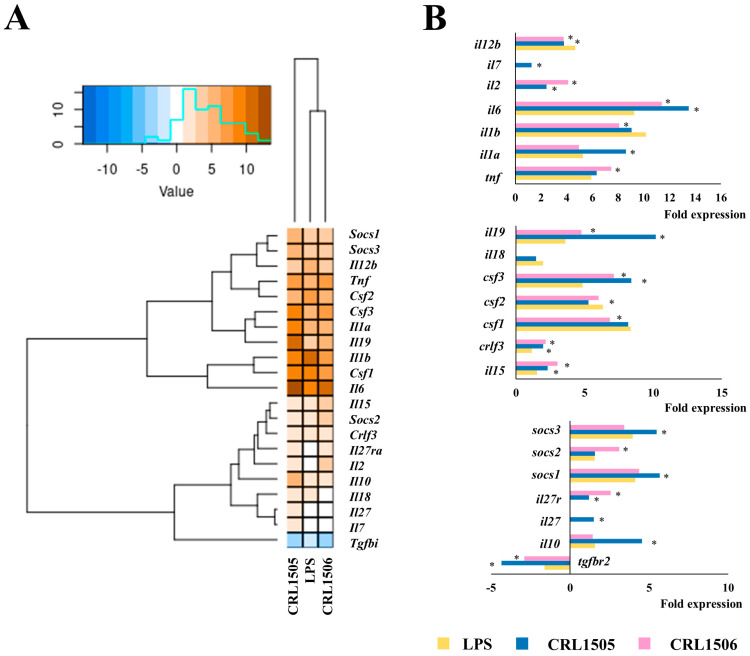
Effect of lactobacilli strains in murine macrophages (RAW cells) stimulated with the Toll-like receptor 4 (TLR4) agonist lipopolysaccharide (LPS). Macrophages were treated with *Lacticaseibacillus rhamnosus* CRL1505 or *Lactiplantibacillus plantarum* CRL1506 strains and then challenged with LPS. The expression of genes was determined by microarray analysis 12 h after LPS stimulation. Non-lactobacilli-treated macrophages stimulated with LPS were used as control. Heatmap analysis (**A**) and fold expression changes (**B**) of cytokines. Asterisks indicate significant differences between the indicated groups and LPS-challenged control macrophages, (*) *p* < 0.05.

The challenge of murine macrophages with LPS significantly increased the expression of the proinflammatory cytokines *tnf* (5.9-fold change), *il1a* (5.2), *il1b* (9.1), *il6* (9.2), *il12* (4.6), *csf1* (6.3), *csf2* (6.3), *csf3* (4.8), *ifnβ1* (6.7), and *ifnγ* (3.4) as well as the regulatory factors *il10* (1.6), *socs1* (4.1), *socs2* (1.6), and *socs3* (3.9) (Figure 3).

No changes in the expression of *il27* were detected while *tgfbr2* was significantly downregulated. When the relative expressions of inflammatory and regulatory cytokines were used to construct a heatmap it was observed that macrophages treated with *L. rhamnosus* CRL1505 have a different expression pattern compared to control and CRL1506-treated macrophages in response to LPS challenge (Figure 3A). Although both lactobacilli increased the expression of *il6*, *il9*, *ifnβ1*, *ifnγ*, and *csf3*, and decreased *il12* and *tgfbr2*, the CRL1505 strain was more efficient to induce this effect than *L. plantarum* CRL1506 (Figure 3B). In addition, only *L. rhamnosus* CRL1505 was able to increase the expression of *il1b*, *il10*, *socs1*, *socs3*, and *il27* and reduce *csf2* compared to control macrophages. On the other hand, only the CRL1506 strain augmented the expression of *tnf*, and *socs2* and reduced *il1b*, and *csf1* compared to control macrophages (Figure 3B). These results show that *L. rhamnosus* CRL1505 is more efficient in modulating cytokine expressions in LPS-stimulated RAW macrophages than *L. plantarum* CRL1506.

#### 2.1.3. The CRL1505 and CRL1506 Strains Modulate Chemokines and Adhesion Molecules in LPS-Stimulated RAW Macrophages

**Figure 4:**
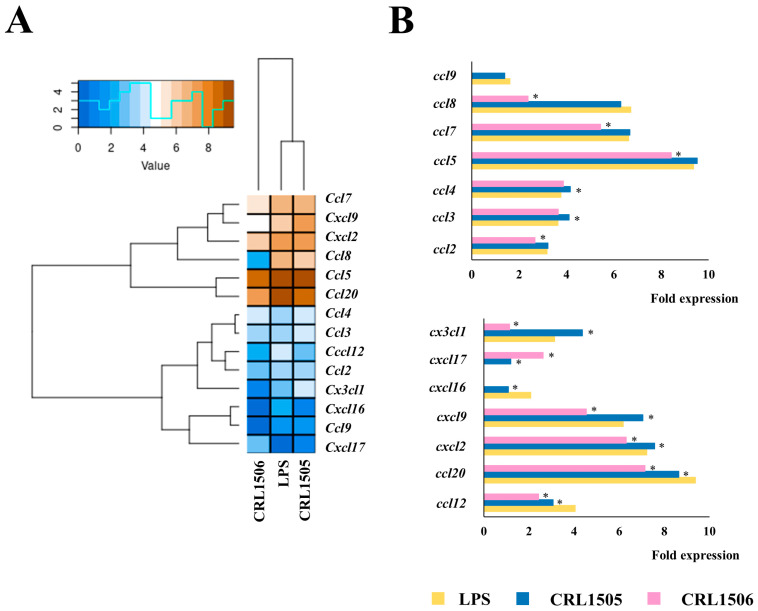
Effect of lactobacilli strains in murine macrophages (RAW cells) stimulated with the Toll-like receptor 4 (TLR4) agonist lipopolysaccharide (LPS). Macrophages were treated with *Lacticaseibacillus rhamnosus* CRL1505 or *Lactiplantibacillus plantarum* CRL1506 strains and then challenged with LPS. The expression of genes was determined by microarray analysis 12 h after LPS stimulation. Non-lactobacilli-treated macrophages stimulated with LPS were used as controls. Heatmap analysis (**A**) and fold expression changes (**B**) of chemokines. Asterisks indicate significant differences between the indicated groups and LPS-challenged control macrophages, (*) *p* < 0.05.

The challenge of murine macrophages with LPS also augmented the expression of the chemokines *ccl2* (3.1-fold change), *ccl4* (3.7), *ccl5* (9.3), *ccl7* (6.6), *ccl8* (6.7), *ccl12* (4.1), *ccl20* (9.4), *cxcl2* (7.2), *cxcl9* (6.1), *cxcl16* (2.1), and *cx3cl1* (3.1) (Figure 4).

Heatmap analysis revealed that both lactobacilli were able to differentially regulate the expressions of chemokines in LPS-challenged macrophages, the effect of *L. rhamnosus* CRL1505 was more remarkable than *L. plantarum* CRL1506 (Figure 4A). Macrophages treated with the CRL1505 strain showed significantly increased expressions of *ccl3* and *ccl4*, while CRL1506-treated macrophages had reduced expressions of *ccl2*, *ccl5*, *ccl7*, and *ccl8* compared to controls (Figure 4B). Both lactobacilli strains reduced *ccl12* and *ccl20* in LPS-challenged macrophages. Of note, *cxcl9*, *cxcl12*, and *cx3cl1* were significantly increased and decreased in macrophages treated with *L. rhamnosus* CRL1505 and *L. plantarum* CRL1506, respectively (Figure 4A). Like cytokines, *L. rhamnosus* CRL1505 is more efficient in modulating chemokine expressions in LPS-stimulated RAW macrophages than *L. plantarum* CRL1506.

**Figure 5:**
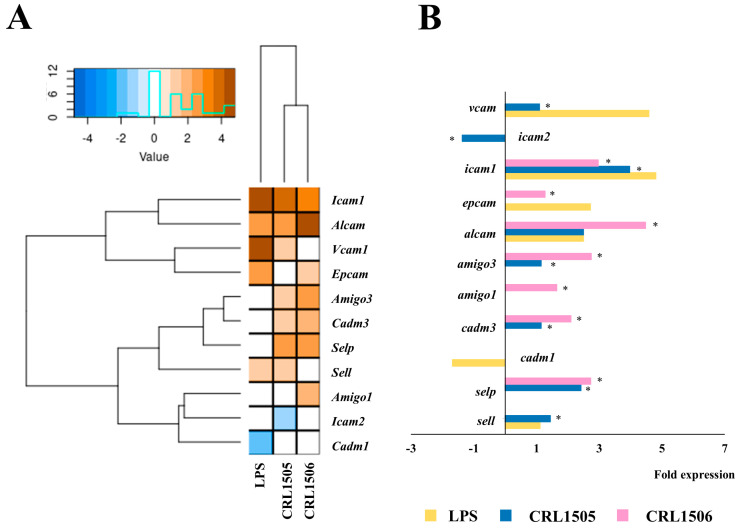
Effect of lactobacilli strains in murine macrophages (RAW cells) stimulated with the Toll-like receptor 4 (TLR4) agonist lipopolysaccharide (LPS). Macrophages were treated with *Lacticaseibacillus rhamnosus* CRL1505 or *Lactiplantibacillus plantarum* CRL1506 strains and then challenged with LPS. The expression of genes was determined by microarray analysis 12 h after LPS stimulation. Non-lactobacilli-treated macrophages stimulated with LPS were used as controls. Heatmap analysis (**A**) and fold expression changes (**B**) of adhesion molecules. Asterisks indicate significant differences between the indicated groups and LPS-challenged control macrophages, (*) *p* < 0.05.

Since both chemokines and adhesion molecules act together to regulate the traffic of immune cells during inflammation, we also evaluated the expression of adhesion molecule genes in LPS-challenged murine macrophages (Figure 5). The activation of TLR4 signaling increased the expression of *alcam* (2.4-fold change), *epcam* (2.9), *icam1* (4.8), *vcam1* (4.5), and *sell* (1.5), and reduced *cadm1* (1.6). Both lactobacilli were able to differentially regulate the expressions of adhesion molecules in LPS-challenged macrophages, but the effect of *L. plantarum* CRL1506 was more remarkable than *L. rhamnosus* CRL1505 (Figure 5A). The CRL1505 strain significantly reduced the expression of *vcam* and *icam2,* and augmented *sell* while the CRL1506 strain increased *amigo1* and reduced *epcam* compared to control macrophages. Both lactobacilli reduced *icam1* and increased *selp* in macrophages after the activation of the TLR4 pathway. In contrast to cytokines and chemokines, *L. rhamnosus* CRL1505 is less efficient in modulating adhesion molecules expressions in LPS-stimulated RAW macrophages than *L. plantarum* CRL1506.

#### 2.1.4. Confirmation of Immune Factors Modulation by CRL1505 and CRL1506 Strains with qPCR

**Figure 6:**
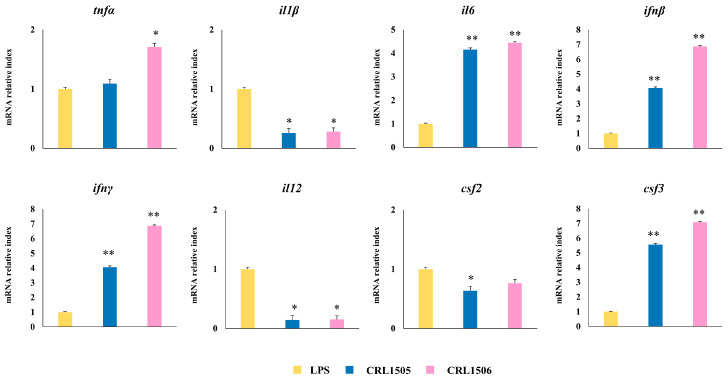
Effect of lactobacilli strains on the expression of cytokines by murine macrophages (RAW cells) stimulated with the Toll-like receptor 4 (TLR4) agonist lipopolysaccharide (LPS). Macrophages were treated with *Lacticaseibacillus rhamnosus* CRL1505 or *Lactiplantibacillus plantarum* CRL1506 strains and then challenged with LPS. The expression of genes was determined by qPCR analysis 12 h after LPS stimulation. Non-lactobacilli-treated macrophages stimulated with LPS were used as controls. Asterisks indicate significant differences between the indicated groups and LPS-challenged control macrophages, (*) *p* < 0.05; (**) *p* < 0.01.

To confirm the changes induced by *L. rhamnosus* CRL1505 and *L. plantarum* CRL1506 on the cytokine expression profile in LPS-challenged murine RAW macrophages, qPCR was performed on selected genes: *tnfα*, *il1β*, *il6*, *ifnβ*, *ifnγ*, *il12*, *csf2*, and *csf3*. As shown in Figure 6, the treatment of macrophages with lactobacilli induced a significant increase in the expression levels of *il6*, *ifnβ*, *ifnγ*, and *csf3* compared to control macrophages. On the other hand, only the CRL1506 strain was able to increase *tnfα* levels compared to controls (Figure 6). Both strains reduced the expression of *il1β* and *il12*, while only *L. rhamnosus* CRL1505 reduced *csf2*.

**Figure 7:**
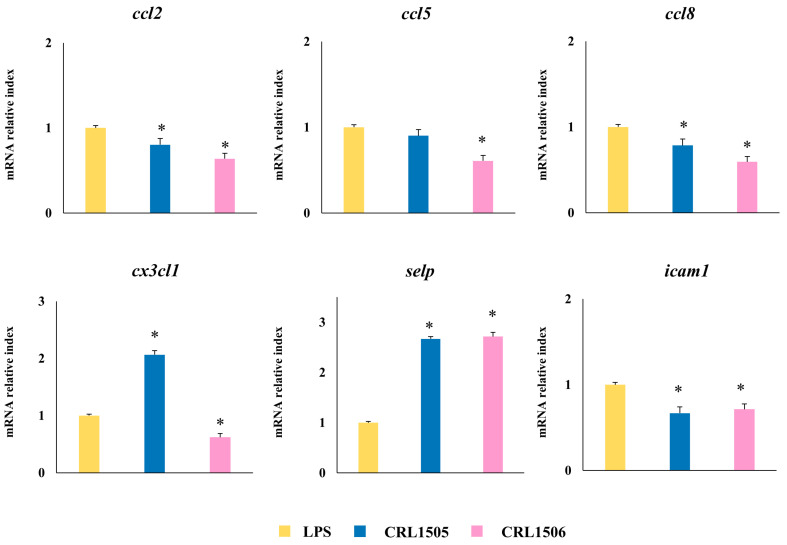
Effect of lactobacilli strains on the expression of chemokines by murine macrophages (RAW cells) stimulated with the Toll-like receptor 4 (TLR4) agonist lipopolysaccharide (LPS). Macrophages were treated with *Lacticaseibacillus rhamnosus* CRL1505 or *Lactiplantibacillus plantarum* CRL1506 strains and then challenged with LPS. The expression of genes was determined by qPCR analysis 12 h after LPS stimulation. Non-lactobacilli-treated macrophages stimulated with LPS were used as controls. Asterisks indicate significant differences between the indicated groups and LPS-challenged control macrophages, (*) *p* < 0.05.

The expressions of the chemokines *ccl2*, *ccl8*, *ccl5*, and *cx3cl1*, as well as the adhesion molecules *icam-1* and *selp* were also analyzed (Figure 7). The treatment of murine macrophages with the lactobacilli strains significantly decreased the expression of *ccl2*, *ccl8*, and *icam1*, and increased *selp* compared to control macrophages after challenge with LPS. The expression level of *cx3cl1* was significantly increased and reduced by *L. rhamnosus* CRL1505 and *L. plantarum* CRL1506 treatments, respectively. In addition, the CRL1506 strain significantly decreased *ccl5* to values below the observed in LPS-challenged control macrophages (Figure 7).

**Figure 8:**
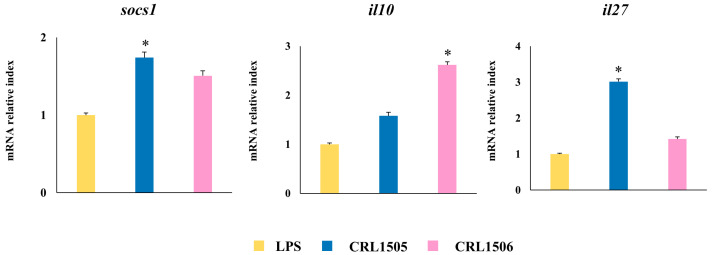
Effect of lactobacilli strains in murine macrophages stimulated with the Toll-like receptor 4 (TLR4) agonist lipopolysaccharide (LPS). Macrophages were treated with *Lacticaseibacillus rhamnosus* CRL1505 or *Lactiplantibacillus plantarum* CRL1506 strains and then challenged with LPS. The expression of genes was determined by qPCR analysis 12 h after LPS stimulation. Non-lactobacilli-treated macrophages stimulated with LPS were used as controls. Asterisks indicate significant differences between the indicated groups and LPS-challenged control macrophages, (*) *p* < 0.05.

When regulatory cytokines expressions were analyzed in macrophages challenged with the TLR4 agonist, it was observed that *L. rhamnosus* CRL1505 upregulated the levels of *socs1* and *il27* compared to macrophages treated with *L. plantarum* CRL1506 and the controls (Figure 8). In addition, the CRL1506 increased the expression of the regulatory cytokine *il10* (Figure 8).

The qPCR results are in line with microarray studies showing that although both immunomodulatory lactobacilli can modify the transcriptomic response of RAW macrophages after LPS stimulation, each strain induces specific immunotranscriptomic changes.

### 2.2. Effect of L. rhamnosus CRL1505 and L. plantarum CRL1506 on Peyer’s Patches APCs and Peritoneal Macrophages Cytokine Production in Response to LPS Stimulation

#### 2.2.1. The CRL1505 and CRL1506 Strains Modulate Cytokine Production in LPS-Stimulated Peyer’s Patches Macrophages

**Figure 9:**
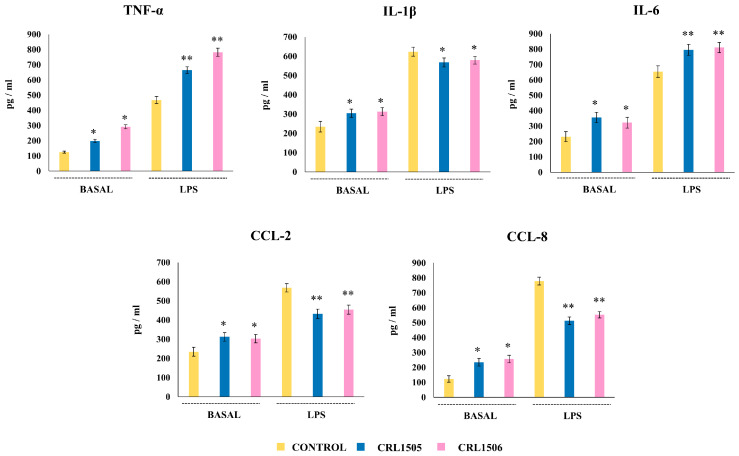
Effect of lactobacilli strains on the production of inflammatory cytokines and chemokines by murine Peyer’s patches macrophages stimulated with the Toll-like receptor 4 (TLR4) agonist lipopolysaccharide (LPS). Immunocompetent adult BALB/c mice (6 weeks) were orally treated with *Lacticaseibacillus rhamnosus* CRL1505 or *Lactiplantibacillus plantarum* CRL1506 strains for five days. On day 6, Peyer’s patches macrophages were isolated, cultured for 24 h, and then challenged with LPS. The levels of cytokines were determined after 24 h of culture (basal) or 24 h after LPS challenge by ELISA. Peyer’s patches of macrophages obtained from non-lactobacilli-treated mice were used as controls. Asterisks indicate significant differences between the indicated groups and basal or LPS-challenged controls, (*) *p* < 0.05; (**) *p* < 0.01.

We next aimed to evaluate in vivo the effect of lactobacilli on the response of macrophages to LPS stimulation. The first set of experiments was performed with macrophages from Peyer’s patches. It was established that within the murine Peyer’s patches phagocytes and macrophages are characterized by a high expression of lysozyme, *cx3cr1*, and *cd4*, which differentiate them from DCs [22,23]. Then, immune cells from Peyer’s patches were isolated and cultured in glass to allow the selection of APCs, followed by magnetic sorting of CD4^+^ cells to obtain macrophages. The levels of TNF-α, IL-6, IL-1β, CCL-2, and CCL-8 in Peyer’s patches macrophages from mice receiving the lactobacilli strains were first analyzed (Figure 9). It was observed that *L. rhamnosus* CRL1505 and *L. plantarum* CRL1506 significantly increased the levels of all the cytokines and chemokines evaluated in non-inflammatory conditions (without LPS challenge). On the other hand, when Peyer’s patches macrophages were cultured and challenged with LPS, an increase in all the cytokines levels was observed with respect to the basal levels in all the experimental groups (Figure 9). However, Peyer’s patches macrophages from mice receiving the lactobacilli strains had significantly increased levels of TNF-α and IL-6 and decreased concentrations of IL-1β, CCL-2, and CCL-8 compared with cells from control mice.

**Figure 10:**
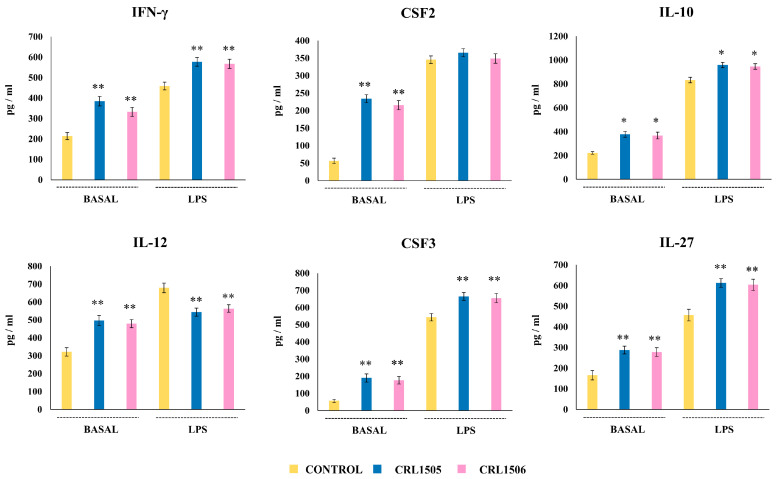
Effect of lactobacilli strains on the production of inflammatory and regulatory cytokines by murine Peyer’s patches macrophages stimulated with the Toll-like receptor 4 (TLR4) agonist lipopolysaccharide (LPS). Immunocompetent adult BALB/c mice (6 weeks) were orally treated with *Lacticaseibacillus rhamnosus* CRL1505 or *Lactiplantibacillus plantarum* CRL1506 strains for five days. On day 6, Peyer’s patches macrophages were isolated, cultured for 24 h, and then challenged with LPS. The levels of cytokines were determined after 24 h of culture (basal) or 24 h after LPS challenge by ELISA. Peyer’s patches macrophages obtained from non-lactobacilli-treated mice were used as controls. Asterisks indicate significant differences between the indicated groups and basal or LPS-challenged controls, (*) *p* < 0.05; (**) *p* < 0.01.

We also evaluated the changes in the levels of IFN-γ, IL-12, CSF2, CSF3, IL-10, and IL-27 in Peyer’s patches macrophages before and after the stimulation with LPS (Figure 10). It was noticed that the basal levels of all these immune factors were significantly higher in lactobacilli-treated macrophages than in controls. When macrophages were stimulated with LPS all the factors increased compared to the basal levels. However, a higher concentration of IFN-γ, CSF3, IL-27, and IL-10 was found in lactobacilli-treated cells than controls (Figure 10). In addition, both *L. rhamnosus* CRL1505 and *L. plantarum* CRL1506 induced a decrease in IL-12 levels. No significant differences were observed in CSF2 values when lactobacilli-treated macrophages were compared to controls. In contrast to the studies in RAW macrophages, the evaluation of cytokines and chemokines in Peyer’s patches macrophages showed that both immunomodulatory lactobacilli induced a similar effect.

#### 2.2.2. The CRL1505 and CRL1506 Strains Modulate Cytokine Production in LPS-Stimulated Peritoneal Macrophages

**Figure 11:**
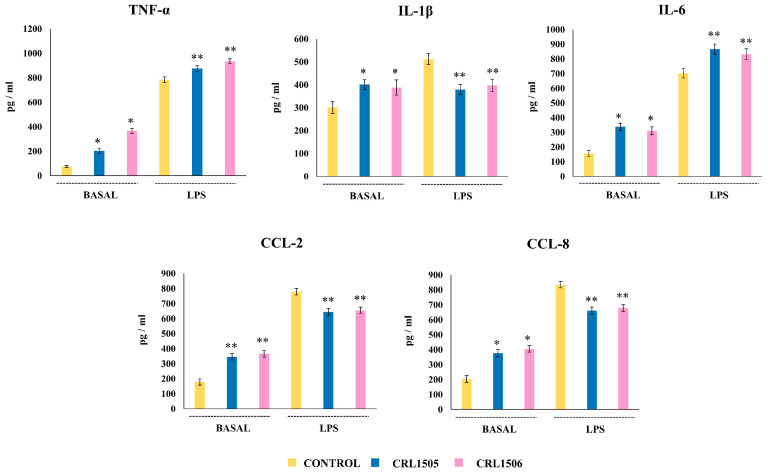
Effect of lactobacilli strains on the production of inflammatory cytokines and chemokines by murine peritoneal macrophages stimulated with the Toll-like receptor 4 (TLR4) agonist lipopolysaccharide (LPS). Immunocompetent adult BALB/c mice (6 weeks) were orally treated with *Lacticaseibacillus rhamnosus* CRL1505 or *Lactiplantibacillus plantarum* CRL1506 strains for five days. On day 6, peritoneal macrophages were isolated, cultured for 24 h, and then challenged with LPS. The levels of cytokines were determined after 24 h of culture (basal) or 24 h after LPS challenge by ELISA. Peritoneal macrophages obtained from non-lactobacilli-treated mice were used as controls. Asterisks indicate significant differences between the indicated groups and basal or LPS-challenged controls, (*) *p* < 0.05; (**) *p* < 0.01.

In a second set of experiments, we isolated peritoneal macrophages from control and lactobacilli-treated mice, stimulated them in vitro with LPS, and evaluated the levels of cytokines (Figure 11). It was shown that the CRL1505 and CRL1506 strains enhanced the basal levels of TNF-α, IL-6, IL-1β, CCL-2, and CCL-8 compared to control mice. The challenge of peritoneal macrophages with LPS increased all the cytokines evaluated compared to basal levels, in all the experimental groups. However, the concentrations of TNF-α and IL-6 were higher in macrophages from mice treated with *L. rhamnosus* CRL1505 and *L. plantarum* CRL1506 than in controls, while IL-1β, CCL-2 and CCL-8 were lower (Figure 11).

**Figure 12:**
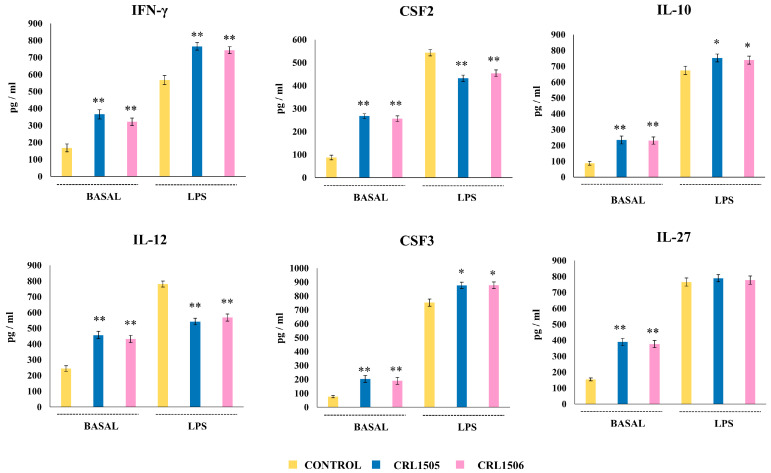
Effect of lactobacilli strains on the production of inflammatory and regulatory cytokines by murine peritoneal macrophages stimulated with the Toll-like receptor 4 (TLR4) agonist lipopolysaccharide (LPS). Immunocompetent adult BALB/c mice (6 weeks) were orally treated with *Lacticaseibacillus rhamnosus* CRL1505 or *Lactiplantibacillus plantarum* CRL1506 strains for five days. On day 6, peritoneal macrophages were isolated, cultured for 24 h, and then challenged with LPS. The levels of cytokines were determined after 24 h of culture (basal) or 24 h after LPS challenge by ELISA. Peritoneal macrophages obtained from non-lactobacilli-treated mice were used as controls. Asterisks indicate significant differences between the indicated groups and basal or LPS-challenged controls, (*) *p* < 0.05; (**) *p* < 0.01.

We detected increases in the basal levels of IFN-γ, IL-12, CSF2, CSF3, IL-10, and IL-27 in peritoneal macrophages from mice fed the lactobacilli strains (Figure 12). All the immune factors increased with respect to their basal levels after the stimulation with LPS. Significant increases of IFN-γ, IL-10, and CSF3 and decreases of IL-12 and CSF2 were observed in peritoneal macrophages from lactobacilli-treated mice compared to controls. Of note, no significant differences were observed in the levels of IL-27 when treated and control peritoneal macrophages were compared (Figure 12).

Again, contrasting the results obtained in RAW macrophages, the evaluation of cytokines and chemokines in peritoneal macrophages showed that both immunomodulatory lactobacilli induced a similar effect.

#### 2.2.3. The CRL1505 and CRL1506 Strains Modulate Adhesion Molecules and Regulatory Factors Expression in LPS-Stimulated Peyer’s Patches and Peritoneal Macrophages

**Figure 13:**
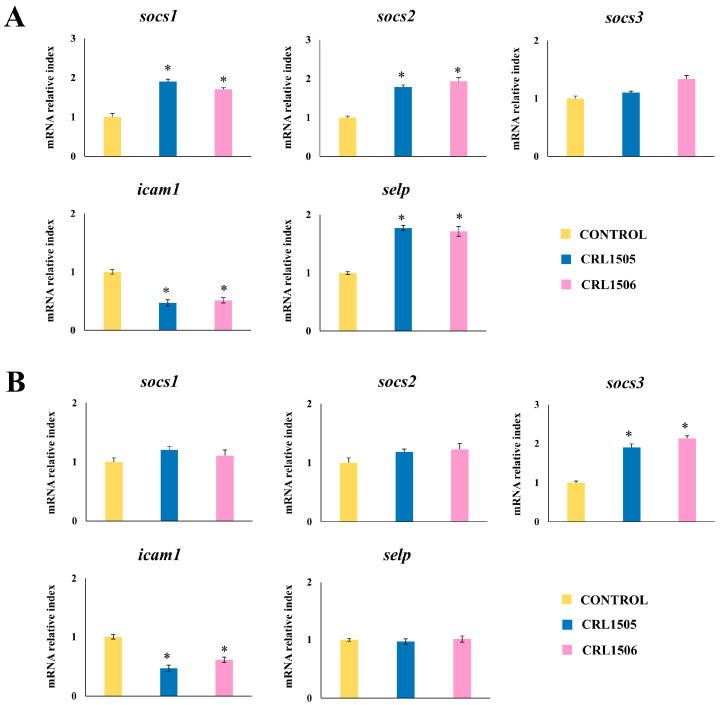
Effect of lactobacilli strains in murine Peyer’s patches and peritoneal macrophages stimulated with the Toll-like receptor 4 (TLR4) agonist lipopolysaccharide (LPS). Immunocompetent adult BALB/c mice (6 weeks) were orally treated with *Lacticaseibacillus rhamnosus* CRL1505 or *Lactiplantibacillus plantarum* CRL1506 strains for five days. On day 6, Peyer’s patches (**A**) and peritoneal (**B**) macrophages were isolated, cultured for 24 h, and then challenged with LPS. The expression of adhesion molecules and socs genes were determined 12 h after LPS challenge by qPCR. Macrophages obtained from non-lactobacilli-treated mice were used as controls. Asterisks indicate significant differences between the indicated groups and LPS-challenged controls, (*) *p* < 0.05.

Finally, we evaluated changes in the expressions of *selp*, *icam1*, *socs1*, *socs2*, and *socs3* in primary cultures of Peyer’s patches and peritoneal macrophages obtained from mice treated with lactobacilli and stimulated with LPS (Figure 13). The expressions of *socs1* and *socs2* were higher in Peyer’s patches macrophages from animals treated with CRL1505 and CRL1506 strains than controls while the lactobacilli did not change *socs3* expression. The opposite was observed in peritoneal macrophages since *L. rhamnosus* CRL1505 and *L. plantarum* CRL1506 only increased *socs3* expression while no changes were detected in *socs1* and *socs2.* In addition, both lactobacilli reduced *icam1* expression in Peyer’s patches and peritoneal macrophages while the upregulation of *selp* was observed only in cells from Peyer’s patches in response to LPS stimulation (Figure 13). Like cytokines and chemokines, the changes in the expressions of regulatory and adhesion factors in Peyer’s patches and peritoneal macrophages induced by both immunomodulatory lactobacilli were similar.

## 3. Discussion

In this work, we aimed to advance in the characterization of the immunomodulatory properties of *L. rhamnosus* CRL1505 and *L. plantarum* CRL1506, focused on the TLR4-triggered immune response in macrophages. These studies were performed to gain insight into the mechanisms involved in their abilities to improve defenses against Gram-negative pathogens, as demonstrated in previous works [19,20,21]. For this purpose, we first used the murine macrophage cell line RAW 264.7 to evaluate the transcriptomic changes induced by the CRL1505 and CRL1506 strains, considering that this in vitro system has been widely used to evaluate immunomodulatory bacteria. Different lactobacilli strains can modulate RAW macrophages’ cytokine profiles in response to TLR4 activation by inducing changes in proinflammatory cytokines and chemokines as well as regulatory factors [12,13,14,15,16]. Although the reduction of inflammatory factor expression in response to LPS challenge has been consistently described in lactobacilli-treated macrophages, the changes in regulatory factors have been more variable. For example, *L. rhamnosus* 4B15 [14] and *L. helveticus* NS8 [15] reduced the expression of inflammatory cytokines in RAW macrophages stimulated with LPS but the 4B15 strain reduced IL-10 while the NS8 strain augmented this regulatory cytokine. Our previous transcriptomic studies with the immunomodulatory strain *L. plantarum* LOC1 detected the reduction of several inflammatory cytokines and chemokines including *il1β*, *il12*, *csf2*, *cxcl3*, *cxcl1*, and *cx3cl1* in RAW macrophages stimulated with LPS [16]. However, we also observed that the LOC1 strain increased *tnfα*, *il6*, *ifnγ*, *il15*, *cxcl9*, and *csf3*, induced no effect on *il10* but augmented *il27* expressions. These results showed that the effect of lactobacilli on the response of macrophages to TLR4 activation is more complex than previously predicted when measuring a limited number of immunological factors. In line with our previous results, microarray analysis and qPCR confirmation showed that the CRL1505- and CRL1506-treated macrophages responded to LPS stimulation with higher *il6*, *il15*, *ifnβ*, *ifnγ*, *csf3*, and *selp* expression and lower *il1β*, *il12*, *ccl2*, *ccl8*, and *icam1* expression compared to control macrophages. Although most of the changes induced by *L. plantarum* CRL1506 and *L. rhamnosus* CRL1505 in the expression of inflammatory cytokines and chemokines and adhesion molecules were similar, the CRL1505 strain induced a more remarkable effect. In addition, changes induced by only one strain were also detected. The CRL1506 strain increased *tnfα* and reduced *cx3cl1*, while the CRL1505 augmented *cx3cl1* and diminished *csf2* and *ccl5.* Of note, changes in anti-inflammatory mediators were also detected in macrophages after the challenge with LPS, and interestingly, lactobacilli strains modulated in a different way these regulatory factors. *L. plantarum* CRL1506 increased *il10* and *socs2* while *L. rhamnosus* CRL1505 upregulated *il27*, *socs1*, and *socs3*. These results show that each strain induces specific immunotranscriptomic changes in RAW macrophages after LPS stimulation, and raise an important question: could the combined use of both strains be more efficient in modulating the response of macrophages and improving resistance to infections? This is an interesting point for future research.

Various immortalized murine macrophage-like cell lines have been used for investigating the role of beneficial microbes on immunity including RAW 264.7, J774A.1, and the peritoneal macrophages PMJ2-R. The use of cell lines avoids the limitations of individual variations observed in primary cultures and the phenotype changes through different passages [24]. However, the response of immortalized cells to bacterial infections or MAMP stimulation may have quantitative or qualitative differences compared to cells of the living organism [25]. In this regard, it was shown that J774A.1 cells stimulated with LPS increased IL-6 and IL-12 production while the stimulation of primary cultures of murine peritoneal macrophages with the same dose of LPS enhanced the production of both cytokines but at levels significantly higher than those observed in J774A.1 macrophages [26]. In a similar approach, it was shown that primary cultures of murine peritoneal macrophages challenged with LPS increased their production of TNF-α and IL-6 while the same dose of the TLR4 agonist augmented these cytokines to a lower extent in RAW 264.7 macrophages [27]. Furthermore, a comparative proteomic study of the PMJ2-R cell line and primary cultures of peritoneal macrophages showed differences in proteins related to the phagocytic process [28]. Then, considering these facts, we aimed to contrast the results of RAW macrophages with those obtained in ex vivo experiments.

Gut macrophages are a heterogeneous population [8]. Within the intestinal tissue, macrophages are distributed at various locations that include the lamina propria, the submucosa, the Peyer’s patches, around the blood vessels, or associated with smooth muscles or neurons. Among these cells, macrophages located in the lamina propria have been extensively studied in terms of their phagocytic, antigen-presenting, and cytokine production capacities because of their close contact with the antigens of the intestinal lumen [29]. These subepithelial macrophages are the first line of contact with intestinal microbes and as such, they possess strong bactericidal and phagocytic properties. However, despite their continuous stimulation by the intestinal microbiota and their MAMPs, subepithelial macrophages do not tend to induce inflammatory responses because of their regulation by IL-10 and IL-25/IL-33 produced by FoxP3^+^ regulatory T cells and IECs, respectively [8,30,31]. Then, subepithelial macrophages are characterized by constitutive expression of IL-10 and no production of inflammatory mediators. In contrast, macrophages from Peyer’s patches are programmed to handle microorganisms differently, particularly pathogens. Peyer’s patches macrophages can produce inflammatory cytokines like TNF-α and IL-6 upon stimulation while they produce low IL-10 compared to subepithelial macrophages [30]. On the other hand, Gram-negative invasive bacteria can damage the intestinal tissue and affect wall integrity translocating to deeper tissues like the peritoneal cavity or reaching the bloodstream. On those tissues, pathogens face different populations of phagocytes. In the peritoneal cavity, the first line of defense is supported by resident large peritoneal macrophages [31] that are F4/80^hi^CD11b^+^MHC-II^−^ and possesses a high phagocytic activity [32] and the ability to respond to pathogens’ MAMPs through the TLR4 and TLR7 pathways [33]. In the steady state, peritoneal macrophages eliminate apoptotic cells avoiding inflammation because of their elevated expression of negative regulators of TLR signaling including *Socs3* and *Tnfaip3* [33]. However, peritoneal macrophages are crucial to orchestrate innate immune responses in front of pathogens as demonstrated by experiments in which their depletion resulted in augmented bacterial load and lower survival of mice infected with different bacteria, including *E. coli* [34]. In fact, peritoneal macrophages stimulated with LPS change the expression of proteins involved in phagocytosis and antigen presentation [35], and release inflammatory chemokines and cytokines promoting phagocyte recruitment to the peritoneal cavity that is crucial for eliminating invading bacteria [36]. Then, considering the important role of Peyer’s patches and peritoneal macrophages in the defense against Gram-negative pathogens, we selected these two populations of immune cells to evaluate ex vivo the effects of the CRL1505 and CRL1506 strains and to compare them with the results obtained in RAW macrophages.

In line with the transcriptomic studies performed in RAW cells, it was observed that Peyer’s patches and peritoneal macrophages obtained from mice orally treated with *L. rhamnosus* CRL1505 and *L. plantarum* CRL1506 and in vitro stimulated with LPS had significantly higher production of TNF-α, IFN-γ, IL-6, and CSF3 as well as lower production of IL-1β, IL-12, CSF2, CCL2 and CCL8. Studies demonstrated that IFN-γ produced by macrophages has a crucial role in the elimination of pathogens including *Salmonella* Typhimurium [37]. In fact, it was reported that the treatment of human macrophages with recombinant IFN-γ improves the internalization and killing of *Salmonella*. The important protective role of IFN-γ against pathogenic *E. coli* was highlighted by studies showing that enterohemorrhagic *E. coli* inhibits the IFN-γ pathway in IECs through the inhibition of STAT-1 phosphorylation, helping the pathogen to evade the immune system [38]. In addition, the neutralization of TNF-α shifts macrophages toward an M2 state and increases the persistence of *Salmonella* Typhimurium in infected mice [39]. The challenge of healthy adult male volunteers with attenuated ETEC prevented intestinal infection upon a secondary challenge with the same bacteria, and this protective effect was associated with improved productions of TNF-α and IL-6 by blood monocytes [40]. Also, CSF3 has been shown to induce the migration of neutrophils which have a key role in bacterial clearance during pathogenic *E. coli* infection [41]. On the other hand, it was demonstrated that inflammatory cytokines and chemokines can play a detrimental role in Gram-negative pathogens infections. Studies performed in murine ileal phosphatidylserine receptor 4 (TIM-4)^+^ macrophages showed that vitamin B12 deficiency upregulated *ccl2*, *cxcl3, cxcl2*, *cxcl10*, *cx3cl1*, *Il1α*, and *il1β* expression as well as *icam1* and *vcam1* [42]. However, despite the higher expression of inflammatory mediators in intestinal macrophages, vitamin B12-deficient mice had higher *Salmonella* Typhimurium loads in the ileal contents and increased spread of the pathogen to the spleen than control animals. Experiments with CSF2^−/−^ and wild-type mice showed that deficiency of CSF2 induced a delay in the onset of inflammatory-mediated tissue damage in the spleens and livers after the infection with *Salmonella* Typhimurium [43]. Comparative studies of wild-type and NLRP3^−/−^ mice showed that after the challenge with LPS or *E. coli* infection NLPR3 deficient animals had significantly lower liver damage, associated with a diminished IL-1β production by macrophages [44]. Like inflammatory cytokines, changes in the expression of adhesion molecules were associated with both beneficial and detrimental effects against pathogens. Our results showed that LPS stimulation increased the expression of *icam1* in all the macrophages used in this study while *selp* was augmented in RAW and Peyer’s patches cells but not modified in peritoneal macrophages. These results are in line with studies showing that peritoneal macrophages challenged ex vivo with LPS showed increases in ICAM-1 [35] while they express P-selectin on the plasma membrane in steady conditions but that the stimulation with LPS or IFN-γ did not alter the levels of this adhesion molecule [45]. Interestingly, *L. rhamnosus* CRL1505 and *L. plantarum* CRL1506 increased *selp* and reduced *icam1* in macrophages challenged with LPS. Studies demonstrated that peritoneal macrophages from young and adult mice differ in the expression of proinflammatory and chemotactic genes, particularly in response to LPS stimulation. Decreased expression of *icam1* and *vcam 1* were found in peritoneal macrophages from adult mice compared to neonatal animals after the activation of TLR4 [46]. Furthermore, a higher influx of inflammatory cells into the peritoneal cavity in neonatal mice than in adult animals was observed after LPS stimulation. These differences between neonates and adults have been associated with neonatal exuberant and detrimental inflammatory reactions. Studies comparing wild-type and SELP^−/−^ mice reported a higher bacterial load and more severe inflammation in SELP-deficient mice after the challenge with *Citrobacter rodentium* [47]. In line with these results, mice lacking functional SELP ligands exacerbate *Salmonella* infection characterized by higher bacterial load and proinflammatory cytokine production [48]. Then, it is tempting to speculate that the effect of *L. rhamnosus* CRL1505 and *L. plantarum* CRL1506 in the balance of the different inflammatory mediators and adhesion molecules would allow improved protection against pathogens together with minimal inflammatory-mediated tissue damage as shown in our in vivo studies with Gram-negative bacterial infections [19,20]. In fact, we observed that *L. rhamnosus* CRL1505 and *L. plantarum* CRL1506 increased the levels of TNF-α, IFN-γ, and IL-6 in response to *Salmonella* Typhimurium [19] and ETEC [20] infections, in line with the results obtained here using LPS as the inflammatory stimulus. It would be interesting to evaluate in those murine models of Gram-negative pathogens infections whether the CRL1505 or CRL1506 strains can also modulate the levels of CSF3, IL-1β, IL-12, CSF2, CCL2, CCL8, and adhesion molecules as we observed in this work.

We also detected that macrophages stimulated with LPS increased their expression of regulatory factors including *il10*, *il27*, *socs1*, *socs2*, and *socs3*. It was reported that the stimulation of macrophages with LPS or the infection with Gram-negative pathogens not only induces changes in their expression and production of inflammatory factors but in addition, modulate regulatory factors like IL-10 and IL-27. Studies performed with RAW macrophages challenged with *Vibrio parahaemolyticus* [49] or *Vibrio vulnificus* [50] and primary cultures of murine peritoneal macrophages infected with *Vibrio harveyi* [51] revealed increments in the production of inflammatory cytokines and chemokines. Interestingly, RAW macrophages infected with *V. parahaemolyticus* had also an improved production of IL-10 compared to uninfected controls [49]. Similarly, murine peritoneal macrophages stimulated with *V. harveyi* upregulated the expression of *il10* [49]. Of note, *L. plantarum* CRL1506 increased *il10* and *socs2* while *L. rhamnosus* CRL1505 upregulated *il27, socs1* and *socs3* in LPS-stimulated RAW macrophages. However, Peyer’s patches macrophages from mice treated with both lactobacilli strains had increased expression of *socs1* and *socs2* as well as higher production of IL-10 and IL-27 than controls. In addition, peritoneal macrophages from mice receiving *L. rhamnosus* CRL1505 or *L. plantarum* CRL1506 had increased *socs3* and IL-10 than macrophages from control animals after LPS stimulation. The differences found between the distinct experimental systems may be due to two reasons. On the one hand, in RAW cells we measured the mRNA expression of *il10* and *il27* while in primary macrophage cultures, the proteins of the regulatory cytokines were determined. However, it should be noted that we evaluated the mRNA expression of *sosc1*, *socs2*, and *socs3* in the three experimental systems and still found differences between them. On the other hand, it is highly feasible that the biology of macrophages from Peyer’s patches and the peritoneal cavity is different from that of the cell line, since in vivo, macrophages have been influenced and programmed by their respective microenvironments. Then, our results show that although RAW cells are useful in vitro systems to evaluate the interaction of microorganisms with the host, the results obtained in this system must be carefully analyzed when extrapolating to other macrophage populations in vivo.

Studies in mice have shown that the modulation of both inflammatory cytokines and regulatory factors by immunomodulatory lactobacilli can improve the protection against pathogens. Orally administered *L. rhamnosus* GG improved the production of IL-12 and IL-10 in macrophages and these changes were associated with enhanced resistance to *Salmonella* infection [52]. Then, we can speculate that the modulation of regulatory factors in macrophages by the CRL1505 and CRL1506 strains would have a protective role in vivo during the course of Gram-negative pathogen infection. Several research works support this statement. SOCS proteins, IL-10 and IL-27 are known to be involved in the control of inflammation [53,54]. Both SOCS1 and SOCS3 regulate TLR4 signaling in macrophages through a negative feedback loop to inhibit cytokine signal transduction [55]. In fact, it was reported that SOCS1^−/−^ mice are highly sensitive to LPS-mediated inflammation. Metabolites with the ability to modulate *socs* expression like the gut microbial ursodeoxycholic acid have been shown to control inflammatory responses in macrophages [56]. The treatment of mice with ursodeoxycholic acid significantly increased *socs1* and *socs3* in macrophages and reduced their expression of *tnfα*, *il1β*, *il6*, and *il12* in response to LPS stimulation. On the other hand, it was demonstrated that prostaglandin I2-dependent induction of IL-10 controls the production of IL-1*β* in peritoneal macrophages upon LPS challenge [57]. Improved production of IL-10 has been associated with reduced intestinal inflammation and protection from ETEC diarrhea [58]. The mucosal administration of IL-27 was reported to exert beneficial effects in murine models of inflammatory bowel disease, through its capacity to modulate macrophage function that regulates T cell activation and IL-10 production [59]. It was also demonstrated in human macrophages that IL-27 suppresses their responses to TNF-α or IL-1β stimulation [60]. Of note, we reported previously increased levels of IL-10 in response to *Salmonella* Typhimurium [19] and ETEC [20] infections in animals treated with the CRL1505 or CRL1506 strains. Then, similar to inflammatory cytokines, it would be of value to determine whether *L. rhamnosus* CRL1505 and *L. plantarum* CRL1506 can modulate the levels of IL-27, *sosc1*, *socs2*, and *socs3* in the murine models of Gram-negative pathogens infections, to determine the precise role of these regulatory factors in the protection against inflammatory-mediated damage.

Another point for future research is to investigate the molecular mechanism(s) used by *L. rhamnosus* CRL1505 and *L. plantarum* CRL1506 to modulate macrophage function. We reported previously that the peptidoglycan and the lipoteichoic acid are key molecules involved in the immunomodulatory effect of the CRL1505 [61] and CRL1506 [62] strains, respectively. In addition, we showed that the modulation of negative regulators of the TLR signaling could be involved in the differential modulation of cytokines and chemokines expression induced by lactobacilli in macrophages [62] and IECs [16,20]. Then, it is tempting to speculate that *L. rhamnosus* CRL1505 through its peptidoglycan, and *L. plantarum* CRL1506 through its lipoteichoic acid interact with PRRs expressed in macrophages inducing changes in the expression of negative regulators that subsequently modify the response of these immune cells to the activation of TLR4. This hypothesis needs to be verified experimentally.

## 4. Materials and Methods

### 4.1. Bacterial Strains

*Lacticaseibacillus rhamnosus* CRL1505 and *Lactiplantibacillus plantarum* CRL1506 belong to the CERELA-CONICET culture collection (Chacabuco 145, San Miguel Tucumán, Argentina) and were originally isolated from goat’s milk from the northwestern region of Argentina [19]. The purified stock cultures were preserved at −80 °C in De man, Rogosa, and Sharpe (MRS) broth (Oxoid, Basingstoke, UK) containing 30% glycerol. Lactobacilli were grown anaerobically twice in MRS broth at 37 °C for 16 h before use. Strains were harvested by centrifugation (800× *g*, 10 min at 4 °C) and washed with sterile phosphate-buffered saline (PBS).

### 4.2. Cell Cultures

The RAW 264.7 mouse macrophage cell line (Riken Cell Bank, Tsukuba, Japan) was used in this study. Murine macrophages were cultured in Dulbecco’s modified eagle medium (DMEM; Thermo Fisher Scientific, Tokyo, Japan) with 10% fetal bovine serum (FBS; Sigma, St. Louis, MO, USA), penicillin (100 U/mL; Thermo Fisher Scientific, Tokyo, Japan) and streptomycin (100 µg/mL; Thermo Fisher Scientific, Tokyo, Japan) in a 5% humidified CO_2_ incubator at 37 °C. RAW 264.7 cells were tested for mycoplasma contamination before use.

### 4.3. Stimulation of Macrophages with Lactobacilli

RAW 264.7 mouse macrophage cells (5 × 10^5^ cells/well) were inoculated in 12-well plates and incubated overnight. Subsequently, the culture medium was changed, and macrophages were stimulated with lactobacilli (5 × 10^7^ cells/mL). For LPS challenge experiments, murine macrophages were cultured as indicated above and stimulated with lactobacilli (5 × 10^7^ cells/mL) for 24 h. Later, wells were washed to eliminate lactobacilli and stimulated with 50 ng/mL LPS (InvivoGen, San Diego, CA, USA) for 12 h. For these experiments, lactobacilli were grown anaerobically in MRS broth at 37 °C for 12 h (log phase).

### 4.4. Microarray

Total RNA was extracted using the RNeasy Mini kit (Qiagen, Tokyo, Japan) as per the manufacturer’s protocol. RNA samples were treated with Qiagen DNAse and the sample integrity was evaluated using an RNA 6000 Nano kit with an Agilent 2100 Bioanalyzer (Agilent Technologies, Santa Clara, CA, USA). Then, cDNA synthesis was performed with 200 ng of RNA. The RNA labeling and hybridization were performed using the SurePrint G3 Mouse GE 8x60K ver2.0 Microarray from Hokkaido System Science Co., Ltd., Sapporo, Japan. Microarray scanning was performed with the Microarray Scanner from Agilent Technologies, while digitalization was done using Agilent Feature Extraction 10.7.3.1.

Data was normalized and gene expression was analyzed by GeneSpring software version 13.1 (Agilent Technologies). Significantly different up- and down-regulated genes within the samples (stimulated with LPS or lactobacilli plus LPS) with respect to control samples (without LPS stimulation) were considered. Two criteria were used for the selection of genes with significant changes in transcript abundance: a cutoff in transcript abundance of at least 2-fold and a *t*-test *p*-value of less than 0.05. The Limma in R software (version 3.2.5) was used for the gene expression statistical analysis. The log_2_ transform ratio was used to express the results. Genes were analyzed using PANTER 11.1 and the Gene Ontology (GO) classification. Microarray data was submitted to NCBI-GEO under the accession number GSE288790.

### 4.5. Quantitative Expression Analysis by qPCR

RNA samples were taken from macrophages before and after the challenge with LPS. Total RNA was extracted with TRIzol Reagent (Invitrogen, Carlsbad, CA, USA). Following the extraction of RNA, the cDNA was prepared utilizing PrimeScript™ RT reagent Kit with gDNA Eraser (Perfect Real Time; Takara Bio, Kusatsu, Shiga, Japan) following the manufacturer’s instructions.

A CFX Connect Real-Time System (BioRad, Hercules, CA, USA) and TB Green Premix Ex Taq II (Takara Bio) were used to perform qPCR. The primers used were described before [60,61]. The amplification conditions were 95 °C for 30 s, 40 cycles at 95 °C for 5 s, and then 60 °C for 30 s. Beta-actin was used as an internal control to normalize mRNA expression.

### 4.6. Animals, Feeding Procedures, and Immune Cell Isolation

This study was carried out in strict accordance with the recommendations of the Guide for the Care and Use of Laboratory Animals of the CERELA, Guide for Animal Experimentation. Five-week-old BALB/c mice were obtained from the closed colony maintained at CERELA (Tucumán, Argentina). They were housed in plastic cages with controlled room temperature (22 ± 2 °C temperature, 55 ± 2% humidity) and mice were fed ad libitum with a conventional balanced diet. Researchers and personnel specialized in animal care and handling at CERELA ensured animal welfare. The health and behavior of the animals were monitored twice a day. The tests for each parameter studied were carried out in 5–6 mice per group. Animals were euthanized immediately after the time point was reached using xylazine and ketamine. No signs of discomfort or pain were observed and there were no deaths before the mice reached the endpoints.

*L. rhamnosus* CRL1505 or *L. plantarum* CRL1506 were administered orally to different groups of mice for five consecutive days at a dose of 10^8^ cells/mouse/day [19]. On the sixth day, the groups treated with the lactobacilli strains and the untreated mice were sacrificed to obtain peritoneal macrophages that were isolated as described previously [21]. For the obtention of macrophages from Peyer’s patches, immune cells were isolated as described before [63] and the total population of antigen-presenting cells (APCs) was first isolated by culturing immune cells on glass and allowing their adherence for 2 h, 37 °C. Adherent cells that include macrophages and dendritic cells (DCs) were then resuspended in DMEM medium and the Dynabeads™ Mouse CD4 Positive Isolation Kit (Invitrogen) and DynaMag™-2 (Invitrogen) were used to obtain macrophages considering their high expression of CD4 [23,24]. The absence of lymphocytes in this cell suspension was confirmed by microscopic analysis.

### 4.7. Cytokine Protein Determinations

Peyer’s patches and peritoneal macrophages from lactobacilli-treated and control mice were isolated, cultured in 12-well plates (5 × 10^5^ cells/well) for 24 h, and then challenged with LPS (50 ng/mL). The levels of cytokines were determined after 24 h of culture (basal) or 24 h after LPS challenge with kits enzyme-linked immunosorbent assay (ELISA) kits following the manufacturer’s recommendations (R&D Systems, Minneapolis, MN, USA). The following ELISA kits were used: TNF-α (Mouse TNF alpha ELISA Kit, High Sensitivity, BMS607-2HS), IL-6 (Mouse IL-6 ELISA Kit, BMS603-2), IL-1β (Mouse IL-1 beta ELISA Kit, BMS6002), IFN-γ (Mouse IFN gamma ELISA Kit, BMS606-2), IL-10 (Mouse IL-10 ELISA Kit, BMS614) and IL-12 (Mouse IL-12 p70 ELISA Kit, BMS6004) from ThermoFisher Scientific (Waltham, MA, USA), and CCL-2 (Mouse CCL2/JE/MCP-1 ELISA Kit Quantikine, MJE00B), CCL-8 (Mouse CCL8/MCP-2 DuoSet ELISA, DY790), IL-27 (Mouse IL-27 p28/IL-30 ELISA Kit Quantikine, M2728), CSF2 (Mouse GM-CSF DuoSet ELISA, DY415), and CSF3 (Mouse G-CSF ELISA Kit Quantikine, MCS00) from R&D Systems (Minneapolis, MN, USA).

### 4.8. Statistical Analysis

All experiments were conducted in triplicate. The results are presented as means with standard deviations. Data distribution was checked for normality prior to statistical analysis. A two-way ANOVA was applied to assess the differences between groups, followed by Tukey’s test for pairwise comparisons. *p* < 0.05 was selected for statistical significance.

## 5. Conclusions

*L. rhamnosus* CRL1505 and *L. plantarum* CRL1506 can improve the protection against *Salmonella* and pathogenic *E. coli* together with minimal inflammatory-mediated tissue damage. In this work, we advanced in the knowledge of the mechanisms involved in this beneficial effect by demonstrating that the CRL1505 and CRL1506 strains can modulate different inflammatory mediators, adhesion molecules, and regulatory factors in macrophages after TLR4 activation, which is a key signaling pathway involved in the defenses against Gram-negative bacterial pathogens. One limitation of our study is that we measured cytokines, chemokines, and adhesion molecules at only one point after LPS challenge in macrophages. To more accurately elucidate the role of each immune factor modulated by *L. rhamnosus* CRL1505 and *L. plantarum* CRL1506 in macrophages, kinetic studies should be performed in the future. Another limitation of our study with Peyer’s patches macrophages is the lack of a single-cell population purification since our methodology probably allowed the primary culture of distinct macrophages. It has been described that macrophages within the Peyer’s patches are a heterogeneous population that includes TIM-4^+^ lysozyme^+^, TIM-4^−^lysozyme^+^, tingible-body (TMB), and dome-associated villus (DAV) macrophages [30]. The TIM-4^−^lysozyme^+^ macrophages were strongly associated with the defense against *Salmonella* Typhimurium in Peyer’s patches [64,65]. These macrophages efficiently recognize intracellular *Salmonella* and trigger the production of IL-1β and IL-18 that mediate protective inflammatory response [23]. On the other hand, our study focused only on one MAPM from Gram-negative pathogens for inducing inflammatory responses in macrophages. Pathogens like *Salmonella* and *E. coli* present several MAMPs to the immune system simultaneously during infections, so different signaling pathways mediated by different receptors control the inflammatory response of immune cells such as macrophages. For example, it has been shown that flagellins derived from *Salmonella* and *E. coli* can induce the activation of TLR5 and NLRC4 pathways, leading to the production of inflammatory cytokines by peritoneal macrophages [66]. Therefore, the study of inflammatory responses triggered by different MAPMs from Gram-negative pathogens on specific populations of intestinal and peritoneal macrophages would be of great relevance for a better understanding of the immunomodulatory effects of the probiotic strains *L. rhamnosus* CRL1505 and *L. plantarum* CRL1506, in the context of bacterial infections.

## Data Availability

The data presented in this study are available throughout the article.

## References

[1] Du L., Lei X., Wang J., Wang L., Zhong Q., Fang X., Li P., Du B., Wang Y., Liao Z. (2022). Lipopolysaccharides Derived from Gram-Negative Bacterial Pool of Human Gut Microbiota Promote Inflammation and Obesity Development. Int. Rev. Immunol..

[2] Locati M., Curtale G., Mantovani A. (2020). Diversity, Mechanisms, and Significance of Macrophage Plasticity. Annu. Rev. Pathol..

[3] Kessel A., Toubi E., Pavlotzky E., Mogilner J., Coran A.G., Lurie M., Karry R., Sukhotnik I. (2008). Treatment with Glutamine Is Associated with Down-Regulation of Toll-like Receptor-4 and Myeloid Differentiation Factor 88 Expression and Decrease in Intestinal Mucosal Injury Caused by Lipopolysaccharide Endotoxaemia in a Rat. Clin. Exp. Immunol..

[4] Mattock E., Blocker A.J. (2017). How Do the Virulence Factors of *Shigella* Work Together to Cause Disease?. Front. Cell. Infect. Microbiol..

[5] Santos R.L., Raffatellu M., Bevins C.L., Adams L.G., Tükel Ç., Tsolis R.M., Bäumler A.J. (2009). Life in the Inflamed Intestine, *Salmonella* Style. Trends Microbiol..

[6] Luo D., Huang Z., Jia G., Zhao H., Liu G., Chen X. (2023). Naringin Mitigates LPS-Induced Intestinal Barrier Injury in Mice. Food Funct..

[7] He Y., Wang D., Liu K., Deng S., Liu Y. (2023). Sodium Humate Alleviates LPS-Induced Intestinal Barrier Injury by Improving Intestinal Immune Function and Regulating Gut Microbiota. Mol. Immunol..

[8] Delfini M., Stakenborg N., Viola M.F., Boeckxstaens G. (2022). Macrophages in the Gut: Masters in Multitasking. Immunity.

[9] Wynn T.A., Chawla A., Pollard J.W. (2013). Macrophage Biology in Development, Homeostasis and Disease. Nature.

[10] Villena J., Kitazawa H. (2014). Modulation of Intestinal TLR4-Inflammatory Signaling Pathways by Probiotic Microorganisms: Lessons Learned from *Lactobacillus jensenii* TL2937. Front. Immunol..

[11] Cristofori F., Dargenio V.N., Dargenio C., Miniello V.L., Barone M., Francavilla R. (2021). Anti-Inflammatory and Immunomodulatory Effects of Probiotics in Gut Inflammation: A Door to the Body. Front. Immunol..

[12] Jung H.S., Lee N.K., Paik H.D. (2024). Heat-Killed *Latilactobacillus sakei* CNSC001WB and *Lactobacillus pentosus* WB693 Have an Anti-Inflammatory Effect on LPS-Stimulated RAW 264.7 Cells. Probiotics Antimicrob. Proteins.

[13] Yoon S., Cho H., Nam Y., Park M., Lim A., Kim J.H., Park J., Kim W. (2022). Multifunctional Probiotic and Functional Properties of *Lactiplantibacillus plantarum* LRCC5314, Isolated from Kimchi. J. Microbiol. Biotechnol..

[14] Oh N.S., Joung J.Y., Lee J.Y., Kim Y. (2018). Probiotic and Anti-Inflammatory Potential of *Lactobacillus rhamnosus* 4B15 and *Lactobacillus gasseri* 4M13 Isolated from Infant Feces. PLoS ONE.

[15] Rong J., Zheng H., Liu M., Hu X., Wang T., Zhang X., Jin F., Wang L. (2015). Probiotic and Anti-Inflammatory Attributes of an Isolate *Lactobacillus helveticus* NS8 from Mongolian Fermented Koumiss. BMC Microbiol..

[16] Suzuki M., Albarracin L., Tsujikawa Y., Fukuyama K., Sakane I., Villena J., Kitazawa H. (2022). *Lactiplantibacillus plantarum* LOC1 Isolated from Fresh Tea Leaves Modulates Macrophage Response to TLR4 Activation. Foods.

[17] Yoda K., He F., Kawase M., Miyazawa K., Hiramatsu M. (2014). Oral Administration of *Lactobacillus gasseri* TMC0356 Stimulates Peritoneal Macrophages and Attenuates General Symptoms Caused by Enteropathogenic *Escherichia coli* Infection. J. Microbiol. Immunol. Infect..

[18] Castillo N.A., de Moreno de Leblanc A., Galdeano C.M., Perdigón G. (2013). Comparative Study of the Protective Capacity against *Salmonella* Infection between Probiotic and Nonprobiotic *Lactobacilli*. J. Appl. Microbiol..

[19] Salva S., Villena J., Alvarez S. (2010). Immunomodulatory Activity of *Lactobacillus rhamnosus* Strains Isolated from Goat Milk: Impact on Intestinal and Respiratory Infections. Int. J. Food Microbiol..

[20] Baillo A., Villena J., Albarracín L., Tomokiyo M., Elean M., Fukuyama K., Quilodrán-Vega S., Fadda S., Kitazawa H. (2022). *Lactiplantibacillus plantarum* Strains Modulate Intestinal Innate Immune Response and Increase Resistance to Enterotoxigenic *Escherichia coli* Infection. Microorganisms.

[21] Marranzino G., Villena J., Salva S., Alvarez S. (2012). Stimulation of Macrophages by Immunobiotic *Lactobacillus* Strains: Influence beyond the Intestinal Tract. Microbiol. Immunol..

[22] Arroyo Portilla C., Fenouil R., Wagner C., Luciani C., Lagier M., Da Silva C., Hidalgo-Villeda F., Spinelli L., Fallet M., Tomas J. (2023). Peyer’s Patch Phagocytes Acquire Specific Transcriptional Programs That Influence Their Maturation and Activation Profiles. Mucosal Immunol..

[23] Bonnardel J., DaSilva C., Henri S., Tamoutounour S., Chasson L., Montañana-Sanchis F., Gorvel J.P., Lelouard H. (2015). Innate and Adaptive Immune Functions of Peyer’s Patch Monocyte-Derived Cells. Cell Rep..

[24] Pamies D., Bal-Price A., Chesné C., Coecke S., Dinnyes A., Eskes C., Grillari R., Gstraunthaler G., Hartung T., Jennings P. (2018). Advanced Good Cell Culture Practice for Human Primary, Stem Cell-Derived and Organoid Models as Well as Microphysiological Systems. ALTEX.

[25] Lee C.Z.W., Kozaki T., Ginhoux F. (2018). Studying Tissue Macrophages in Vitro: Are IPSC-Derived Cells the Answer?. Nat. Rev. Immunol..

[26] Hung Y.L., Wang S.C., Suzuki K., Fang S.H., Chen C.S., Cheng W.C., Su C.C., Yeh H.C., Tu H.P., Liu P.L. (2019). Bavachin Attenuates LPS-Induced Inflammatory Response and Inhibits the Activation of NLRP3 Inflammasome in Macrophages. Phytomedicine.

[27] He G., Zhang X., Chen Y., Chen J., Li L., Xie Y. (2017). Isoalantolactone Inhibits LPS-Induced Inflammation via NF-ΚB Inactivation in Peritoneal Macrophages and Improves Survival in Sepsis. Biomed. Pharmacother..

[28] Rusanov A.L., Kozhin P.M., Tikhonova O.V., Zgoda V.G., Loginov D.S., Chlastáková A., Selinger M., Sterba J., Grubhoffer L., Luzgina N.G. (2021). Proteome Profiling of PMJ2-R and Primary Peritoneal Macrophages. Int. J. Mol. Sci..

[29] Ning S., Zhang Z., Zhou C., Wang B., Liu Z., Feng B. (2024). Cross-Talk between Macrophages and Gut Microbiota in Inflammatory Bowel Disease: A Dynamic Interplay Influencing Pathogenesis and Therapy. Front. Med..

[30] Wagner C., Bonnardel J., Da Silva C., Martens L., Gorvel J.P., Lelouard H. (2018). Some News from the Unknown Soldier, the Peyer’s Patch Macrophage. Cell. Immunol..

[31] Ardavín C., Alvarez-Ladrón N., Ferriz M., Gutiérrez-González A., Vega-Pérez A. (2023). Mouse Tissue-Resident Peritoneal Macrophages in Homeostasis, Repair, Infection, and Tumor Metastasis. Adv. Sci..

[32] Gautiar E.L., Shay T., Miller J., Greter M., Jakubzick C., Ivanov S., Helft J., Chow A., Elpek K.G., Gordonov S. (2012). Gene-Expression Profiles and Transcriptional Regulatory Pathways That Underlie the Identity and Diversity of Mouse Tissue Macrophages. Nat. Immunol..

[33] Roberts A.W., Lee B.L., Deguine J., John S., Shlomchik M.J., Barton G.M. (2017). Tissue-Resident Macrophages Are Locally Programmed for Silent Clearance of Apoptotic Cells. Immunity.

[34] Vega-Pérez A., Villarrubia L.H., Godio C., Gutiérrez-González A., Feo-Lucas L., Ferriz M., Martínez-Puente N., Alcaín J., Mora A., Sabio G. (2021). Resident Macrophage-Dependent Immune Cell Scaffolds Drive Anti-Bacterial Defense in the Peritoneal Cavity. Immunity.

[35] Yang L., Gong T., Shen H., Pei J., Zhang L., Zhang Q., Huang Y., Hu Z., Pan Z., Yang P. (2021). Precision N-Glycoproteomic Profiling of Murine Peritoneal Macrophages After Different Stimulations. Front. Immunol..

[36] Bain C.C., Jenkins S.J. (2018). The Biology of Serous Cavity Macrophages. Cell Immunol..

[37] Gordon M.A., Jack D.L., Dockrell D.H., Lee M.E., Read R.C. (2005). Gamma Interferon Enhances Internalization and Early Nonoxidative Killing of *Salmonella* Enterica Serovar *typhimurium* by Human Macrophages and Modifies Cytokine Responses. Infect. Immun..

[38] Ho N.K., Ossa J.C., Silphaduang U., Johnson R., Johnson-Henry K.C., Sherman P.M. (2012). Enterohemorrhagic *Escherichia coli* O157:H7 Shiga Toxins Inhibit Gamma Interferon-Mediated Cellular Activation. Infect. Immun..

[39] Pham T.H.M., Brewer S.M., Thurston T., Massis L.M., Honeycutt J., Lugo K., Jacobson A.R., Vilches-Moure J.G., Hamblin M., Helaine S. (2020). *Salmonella*-Driven Polarization of Granuloma Macrophages Antagonizes TNF-Mediated Pathogen Restriction during Persistent Infection. Cell Host Microbe.

[40] Porbahaie M., van den Belt M., Ulfman L., Ruijschop R.M.A.J., Lucas-Van de Bos E., Hartog A., Lenz S., van Alen-Boerrigter I.J., Teodorowicz M., Savelkoul H.F.J. (2023). Low Doses of Diarrhoeagenic *E. coli* Induce Enhanced Monocyte and MDC Responses and Prevent Development of Symptoms after Homologous Rechallenge. PLoS ONE.

[41] Ingersoll M.A., Kline K.A., Nielsen H.V., Hultgren S.J. (2008). G-CSF Induction Early in Uropathogenic *Escherichia coli* Infection of the Urinary Tract Modulates Host Immunity. Cell. Microbiol..

[42] Ge Y., Zadeh M., Sharma C., Lin Y.D., Soshnev A.A., Mohamadzadeh M. (2024). Controlling Functional Homeostasis of Ileal Resident Macrophages by Vitamin B12 during Steady State and *Salmonella* Infection in Mice. Mucosal Immunol..

[43] Coon C., Beagley K.W., Bao S. (2009). The Role of Granulocyte Macrophage-Colony Stimulating Factor in Gastrointestinal Immunity to Salmonellosis. Scand. J. Immunol..

[44] Shen Y., Gong Z., Zhang S., Cao J., Mao W., Yao Y., Zhao J., Li Q., Liu K., Liu B. (2023). Besides TLR2 and TLR4, NLRP3 Is Also Involved in Regulating *Escherichia coli* Infection-Induced Inflammatory Responses in Mice. Int. Immunopharmacol..

[45] Tchernychev B., Furie B., Furie B.C. (2003). Peritoneal Macrophages Express Both P-Selectin and PSGL-1. J. Cell Biol..

[46] Winterberg T., Vieten G., Feldmann L., Yu Y., Hansen G., Hennig C., Ure B.M., Kuebler J.F. (2014). Neonatal Murine Macrophages Show Enhanced Chemotactic Capacity upon Toll-like Receptor Stimulation. Pediatr. Surg. Int..

[47] Kum W.W.S., Lo B.C., Deng W., Ziltener H.J., Finlay B.B. (2010). Impaired Innate Immune Response and Enhanced Pathology during *Citrobacter rodentium* Infection in Mice Lacking Functional P-Selectin. Cell. Microbiol..

[48] Kum W.W.S., Lee S., Grassl G.A., Bidshahri R., Hsu K., Ziltener H.J., Finlay B.B. (2009). Lack of Functional P-Selectin Ligand Exacerbates *Salmonella* Serovar *typhimurium* Infection. J. Immunol..

[49] Waters S., Luther S., Joerger T., Richards G.P., Boyd E.F., Parent M.A. (2013). Murine Macrophage Inflammatory Cytokine Production and Immune Activation in Response to *Vibrio parahaemolyticus* Infection. Microbiol. Immunol..

[50] Qin K., Fu K., Liu J., Wu C., Wang Y., Zhou L. (2019). *Vibrio vulnificus* Cytolysin Induces Inflammatory Responses in RAW264.7 Macrophages through Calcium Signaling and Causes Inflammation in Vivo. Microb. Pathog..

[51] Yu G., Wang J., Zhang W., Yang Q., Liu G., Wang L., Bello B.K., Zhang X., Zhang T., Fan H. (2021). NLRP3 Inflammasome Signal Pathway Involves in *Vibrio harveyi*-Induced Inflammatory Response in Murine Peritoneal Macrophages in Vitro. Acta Biochim. Biophys. Sin..

[52] Duan B., Shao L., Liu R., Msuthwana P., Hu J., Wang C. (2021). *Lactobacillus rhamnosus* GG Defense against *Salmonella* Enterica *Serovar typhimurium* Infection through Modulation of M1 Macrophage Polarization. Microb. Pathog..

[53] Wormald S., Hilton D.J. (2004). Inhibitors of Cytokine Signal Transduction. J. Biol. Chem..

[54] De Oca M.M., De Labastida Rivera F., Winterford C., Frame T.C.M., Ng S.S., Amante F.H., Edwards C.L., Bukali L., Wang Y., Uzonna J.E. (2020). IL-27 Signalling Regulates Glycolysis in Th1 Cells to Limit Immunopathology during Infection. PLoS Pathog..

[55] Sobah M.L., Liongue C., Ward A.C. (2021). SOCS Proteins in Immunity, Inflammatory Diseases, and Immune-Related Cancer. Front. Med..

[56] Fathima A., Jamma T. (2024). UDCA Ameliorates Inflammation Driven EMT by Inducing TGR5 Dependent SOCS1 Expression in Mouse Macrophages. Sci. Rep..

[57] Ipseiz N., Pickering R.J., Rosas M., Tyrrell V.J., Davies L.C., Orr S.J., Czubala M.A., Fathalla D., Robertson A.A., Bryant C.E. (2020). Tissue-Resident Macrophages Actively Suppress IL-1beta Release via a Reactive Prostanoid/IL-10 Pathway. EMBO J..

[58] Brubaker J., Zhang X., Bourgeois A.L., Harro C., Sack D.A., Chakraborty S. (2021). Intestinal and Systemic Inflammation Induced by Symptomatic and Asymptomatic Enterotoxigenic *E. coli* Infection and Impact on Intestinal Colonization and ETEC Specific Immune Responses in an Experimental Human Challenge Model. Gut Microbes.

[59] Andrews C., McLean M.H., Hixon J.A., Pontejo S.M., Starr T., Malo C., Cam M., Ridnour L., Hickman H., Steele-Mortimer O. (2023). IL-27 Induces an IFN-like Signature in Murine Macrophages Which in Turn Modulate Colonic Epithelium. Front. Immunol..

[60] Kalliolias G.D., Gordon R.A., Ivashkiv L.B. (2010). Suppression of TNF-α and IL-1 Signaling Identifies a Mechanism of Homeostatic Regulation of Macrophages by IL-27. J. Immunol..

[61] Clua P., Tomokiyo M., Raya Tonetti F., Islam M.A., García Castillo V., Marcial G., Salva S., Alvarez S., Takahashi H., Kurata S. (2020). The Role of Alveolar Macrophages in the Improved Protection against Respiratory Syncytial Virus and Pneumococcal Superinfection Induced by the Peptidoglycan of *Lactobacillus rhamnosus* CRL1505. Cells.

[62] Mizuno H., Arce L., Tomotsune K., Albarracin L., Funabashi R., Vera D., Islam M.A., Vizoso-Pinto M.G., Takahashi H., Sasaki Y. (2020). Lipoteichoic Acid Is Involved in the Ability of the Immunobiotic Strain *Lactobacillus plantarum* CRL1506 to Modulate the Intestinal Antiviral Innate Immunity Triggered by TLR3 Activation. Front. Immunol..

[63] Garcia-Castillo V., Tomokiyo M., Raya Tonetti F., Islam M.A., Takahashi H., Kitazawa H., Villena J. (2020). Alveolar Macrophages Are Key Players in the Modulation of the Respiratory Antiviral Immunity Induced by Orally Administered *Lacticaseibacillus rhamnosus* CRL1505. Front. Immunol..

[64] Da Silva C., Wagner C., Bonnardel J., Gorvel J.P., Lelouard H. (2017). The Peyer’s Patch Mononuclear Phagocyte System at Steady State and during Infection. Front. Immunol..

[65] Lelouard H., Henri S., De Bovis B., Mugnier B., Chollat-Namy A., Malissen B., Méresse S., Gorvel J.P. (2010). Pathogenic Bacteria and Dead Cells Are Internalized by a Unique Subset of Peyer’s Patch Dendritic Cells That Express Lysozyme. Gastroenterology.

[66] Yang J., Zhang E., Liu F., Zhang Y., Zhong M., Li Y., Zhou D., Chen Y., Cao Y., Xiao Y. (2014). Flagellins of *Salmonella typhi* and Nonpathogenic *Escherichia coli* Are Differentially Recognized through the NLRC4 Pathway in Macrophages. J. Innate Immun..

